# Chainsaw-Related Extremity Injuries

**DOI:** 10.3390/medicina61040759

**Published:** 2025-04-20

**Authors:** Hüseyin Utku Özdeş, Emre Ergen, Muhammed Köroğlu, Mustafa Karakaplan, Ömer Acet, Fırat Al, İdris Çoban, Ferdi Özdemir, Oğuzhan Tok, Tahsin Sarıbas, Harun Köse, Şeyma Yaşar, Kadir Ertem, Okan Aslantürk

**Affiliations:** 1 Department of Orthopedic and Trauma Surgery, Faculty of Medicine, Inonu University, 44280 Malatya, Türkiye; dr.utkuozdes@gmail.com (H.U.Ö.); emre.ergen@inonu.edu.tr (E.E.); muhammed.koroglu@inonu.edu.tr (M.K.); karakaplanmb@gmail.com (M.K.); omer.acet@inonu.edu.tr (Ö.A.); firat.al@inonu.edu.tr (F.A.); idriscoban21@hotmail.com (İ.Ç.); drferdiozdemir@gmail.com (F.Ö.); oguzhan.tok@inonu.edu.tr (O.T.); tahsin.saribas@inonu.edu.tr (T.S.); harunkose925@hotmail.com (H.K.); kadir.ertem@inonu.edu.tr (K.E.); 2 Department of Biostatistics and Medical Informatics, Faculty of Medicine, Inonu University, 44280 Malatya, Türkiye; seyma.yasar@inonu.edu.tr

**Keywords:** chainsaw, rural areas, seasonal variation, work time loss, bone chipping

## Abstract

*Background:* Chainsaw-related injuries vary from skin lacerations to amputation and may cause serious loss of work time in addition to temporary or permanent disability. Most studies in the literature have reported injuries to the lower or upper extremities separately. The aim of our study is to compare the loss of work time between upper- and lower-extremity chainsaw-related non-occupational injuries in rural areas. *Methods:* Chainsaw-related injuries that occurred in rural areas and were treated in our center between 2012 and 2022 were retrospectively reviewed. The patients’ demographics, the injured side and structures, the hand dominance of operators, lengths of hospital stays, the numbers of operations, complications, and loss of work time were recorded. *Results:* In total, 185 patients (181 males and four females) were enrolled in this study. The mean age was 45.5 years (range: 17–81). The mean follow-up time was 9.3 months (range: 6–24). The lower extremities were affected in 109 patients, while the upper extremities were affected in 76. The loss of work time was 60 and 75 days for lower- and upper-extremity injuries, respectively, and was statistically significantly higher for upper-extremity injuries (*p* < 0.001). The fracture rate was higher in the upper than the lower extremities, at 50% and 26.6%, respectively. *Conclusions*: Chainsaws may cause severe injuries in both the upper and lower extremities, and while the lower extremities were affected more frequently, upper-extremity injuries caused a greater loss of work time. Through the use of protective gear and simple precautions, chainsaw-related injuries and the associated loss of work time can be prevented.

## 1. Introduction

The chainsaw is a wood-cutting power tool commonly used by both professional and non-professional users. Although chainsaws can cause serious soft tissue, neurovascular, and bone injuries, chainsaw-related injuries are relatively rarely reported in the literature [1]. Previous studies have usually reported upper- and lower-extremity injuries separately [1,2,3], while studies investigating both the upper and lower extremities have reported a limited number of patients without a detailed description of the associated injuries [4,5,6]. Chainsaw-related injuries may cause tendon and neurovascular injuries, bone fractures, and even amputations [1,2,3,4,7]. These injuries can also cause a serious loss of work time [1,6,7].

The existing literature on saw injuries has commonly focused on table-saw-related injuries [8,9,10,11], which mostly affect the upper extremities [8,9,10,11]. In contrast, chainsaws may affect all appendages due to the associated mechanisms of injury. Kickback injuries may affect the head, face, or upper extremities [12]. While slipping off the wood during chopping wood causes lower-extremity injuries, upper-extremity injuries are generally caused by a direct hit to the appendage holding the wood.

In the existing literature, a limited number of studies have reported the loss of work time or impairment of daily activities related to chainsaw-related injuries, and none of the previous relevant studies have compared the loss of work time between those to the upper and lower extremities. We hypothesized that upper-extremity injuries cause more loss of work time than lower-extremity injuries. In the current study, we aimed to report detailed data (patient demographics, seasonal changes, injury patterns, and treatments) relating to chainsaw-related injuries from rural areas treated at a Level 1 trauma center over a period of 10 years, comparing the loss of work time incurred by upper- and lower-extremity injuries.

## 2. Materials and Methods

After obtaining approval from our university’s ethical committee (2021/2741), we retrospectively reviewed patient files dating from 1 January 2012 to 31 December 2021. The patients treated for chainsaw-related extremity injuries in the department of orthopedics and traumatology were recorded. Informed consent was obtained from all patients.

Patients from rural areas who had been injured with a chainsaw—in particular, with non-occupational injuries—and treated by the orthopedic and traumatology department, with complete data and at least 6 months of follow-up, were included in this study. Patients with injuries due to factors other than chainsaws, occupational injuries, incomplete data, and follow-up times shorter than 6 months were excluded from this study.

The demographics of patients, the type of injury, the injured extremities and structures, the affected side, hand dominance, the number of performed surgeries, complications, lengths of stay (LOS), and loss of work time were analyzed.

After physical and radiological (plain radiographs and Doppler ultrasonography, if required) examinations, tetanus vaccines and intravenous antibiotic (cephazolin) treatment for prophylaxis were administered.

All patients treated in the operating theater—under local, general, spinal (for lower-extremity injuries), or axillary block (for upper-extremity injuries) anesthesia—had their wounds debrided radically. Clothing pieces were often found in the wound, especially in cases of lower-extremity injuries. Fractures of the hand and foot bones were treated with K-wire fixation. Long bones (e.g., the fibula) were initially treated with debridement, and fixation was performed later after it had been decided that the wound was clean. Tendons and muscle injuries were repaired primarily. If present, arterial and nerve injuries were treated by an experienced microsurgeon. Cast splints were applied to patients treated for fractures and tendon, nerve, and vascular injuries.

After surgery, cefazolin, gentamycin, and metronidazole were administered. Low-molecular-weight heparin or aspirin was administered for thromboembolic prophylaxis if needed.

### Sample Size and Statistical Analyses

In this study, with the aim of comparing the loss of labor associated with chainsaw-related lower- and upper-extremity injuries treated in a Level 1 trauma center in a rural area over a period of 10 years, according to the theoretical power analysis performed using the G*Power 3.1 program, the type I error amount (alpha) was 0.05, the power of the test (1-beta) was 0.80, the effect size (d) was 0.46 (medium), and the minimum sample size required to find a significant difference with the alternative hypothesis (H1) was calculated as 76 in each group and for 152 patients in total (Kaynak: Buchner A, Erdfelder E, Faul F, Lang A. G* Power 3.1 manual. Düsseldorf, Germany: Heinrich-Heine-Universitat Dusseldorf. 2017). The presented data are summarized as the mean ± standard deviation (SD) and median (min–max). Conformity to the normal distribution was assessed using the Kolmogorov–Smirnov test. The Mann–Whitney U-test and independent-samples *t*-test were used, where appropriate, for statistical analysis. A *p*-value less than 0.05 was considered statistically significant. IBM SPSS Statistics 28.0 (SPSS Inc., Chicago, IL, USA) was used for the analysis.

## 3. Results

A total of 185 patients were included in our study. The demographic data of the patients are given in Table 1. The left side was more commonly affected, making up 79.8% and 76.3% of lower- and upper-extremity injuries, respectively. The mean length of stay (LOS) was 2.13 days (range from 1–10) and 2.39 days (range from 1–15) for lower-extremity and upper-extremity injuries, respectively. The mean number of operations was statistically significantly higher in the upper extremity group (*p* = 0.004): 1.01 (range, 1–2), and 1.17 (range, 1–3) for lower- and upper-extremity injuries, respectively (Table 2). The loss of work time was statistically significantly higher in the upper extremity group (*p* < 0.001) and was 57.89 and 75.19 days for lower- and upper-extremity injuries, respectively (Table 2). All patients except for one from the upper-extremity injury group were able to return to their previous work (99.4%).

**Figure 1:**
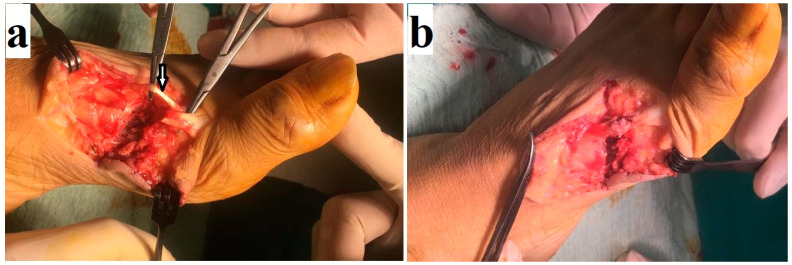
Extensor hallucis longus (EHL) rupture and chipping of the first metatarsal caused by a chainsaw (**a**). Note that the extensor hallucis brevis (arrow) was intact and that the EHL was primarily repaired (**b**).

**Figure 2:**
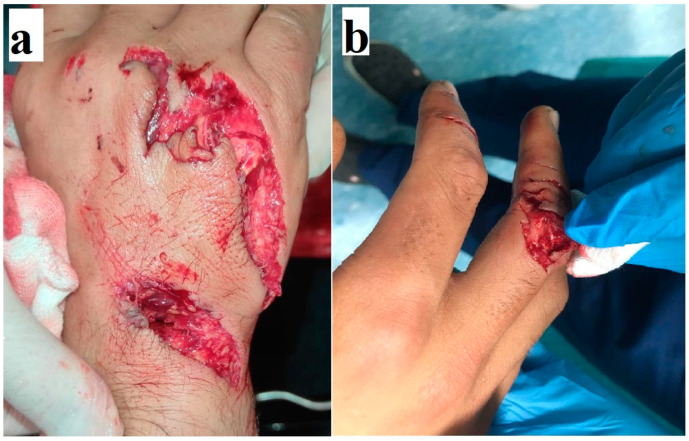
Right-hand dorsal-side injury caused by a chainsaw (**a**). X-ray showing metacarpal fractures (**b**).

The distal and medial sides of the extremity were most commonly affected in the lower extremities, with the extensor hallucis longus (EHL) being the most commonly affected anatomical structure (Figure 1). Meanwhile, in the upper extremities, extensor tendons were the most commonly affected (Figure 2). Detailed information regarding the lower- and upper-extremity injuries is presented in Table 3 and Table 4.

In 19 of the lower extremity patients, there were only skin lacerations; in these cases, debridement and primary closure of the wound were performed. Three patients presented with toe amputations; two of them were not suitable for replantation and underwent primary stump closure. Replantation was performed for the great toe in one patient. In the upper extremities, eight patients presented with total finger amputation, of which seven underwent primary stump closure. One patient had multiple finger amputations (second, third, and fourth fingers); replantation was performed for the second and third fingers, while stump closure was performed for the fourth finger. The patient was not able to return to previous work after the injury resulted in permanent disability.

**Figure 3:**
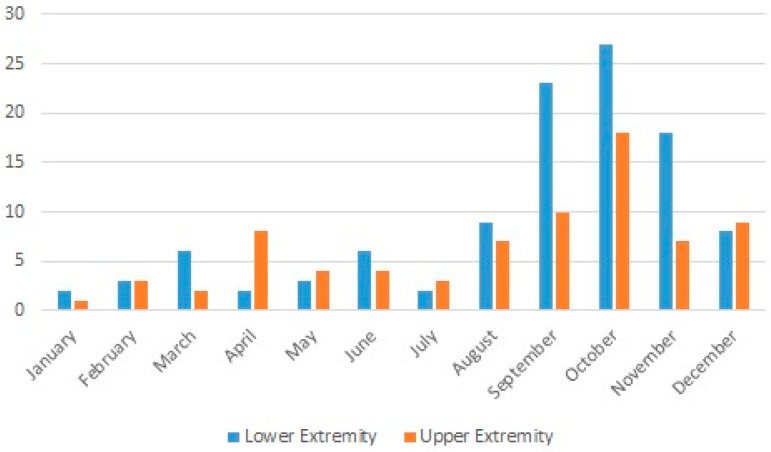
Distribution of lower- and upper-extremity injuries by month.

**Figure 4:**
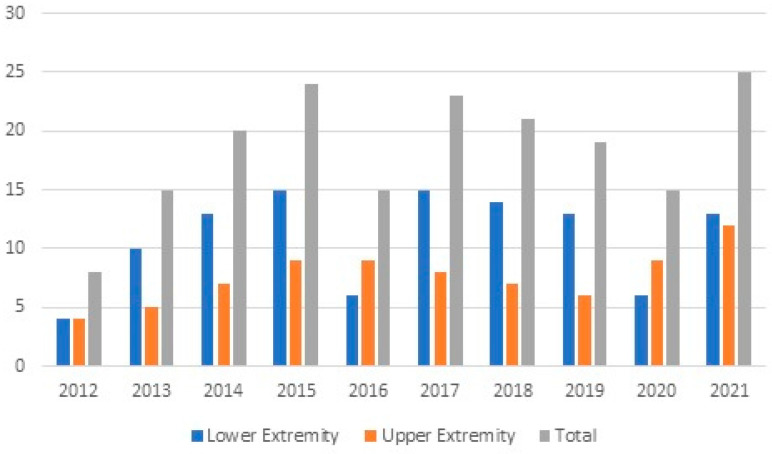
Distribution of lower- and upper-extremity injuries by year.

The fracture rate was higher in the upper than lower extremities, with rates of 50% (42 fractures in 38 patients) and 26.6% (29 fractures in 29 patients), respectively (Table 3 and Table 4). The distribution of injuries by season is given in Table 1. Injuries were most commonly observed in October (n: 45) and were less common in January (n: 3) (Figure 3). There was no statistically significant difference between lower- and upper-extremity injuries when compared by month (*p* = 0.139). The distribution of injuries by year is also shown in Figure 4.

Complications occurred in 12 patients with lower-extremity injuries. Ten patients had superficial wound infections, which were treated with antibiotics. One patient underwent reoperation for EHL re-rupture and one patient underwent great toe amputation after replantation failure. Five superficial wound infections were seen in upper-extremity injuries treated with antibiotics. Tenolysis was performed in four patients. Three patients underwent debridement due to skin necrosis. Amputation was performed in one patient. An autologous bone graft was performed for proximal phalanx non-union in one patient.

## 4. Discussion

Saw-related injuries to the extremities may cause various injuries, varying from skin lacerations to appendage loss [1,2]. The hand has been reported as the most affected appendage [8]. However, lower-extremity injuries have also been reported. The vast majority of saw-related injuries affect the distal parts of the upper and lower extremities. In the current study, the incidence of lower-extremity injuries was higher than those in the upper extremities. The dorsomedial area of the forefoot was the most affected area and the EHL tendon was the most affected structure. To the best of our knowledge, no previous study has compared the loss of work time between lower- and upper-extremity injuries. In our study, the loss of work time for upper-extremity injuries was statistically significantly higher than that for lower-extremity injuries.

Loss of work time due to chainsaw injuries has been rarely reported in the literature [1,6]. In these studies, the authors reported only lower- or upper-extremity injuries. In the current study, we compared the effects of chainsaw injuries in the lower and upper extremities. Loss of work time and the number of operations were statistically significantly higher in patients with upper-extremity injuries (Table 2). Hoxie et al. have reported a mean of 64 days of work time loss after saw-related hand injuries [6]. In our study, the loss of work time due to upper-extremity injuries was 75.19 days. These differences may be due to Hoxie et al. including all saw injuries, including those from table saws and electric bandsaws. In the current study, all patients were injured with a chainsaw, which can cause more serious injuries than table saws. The cutting chain consists of a series of small blades approximately one-quarter to three-eighths of an inch wide, which are depth-controlled and cut in a series of chipping actions. This can not only cause fractures and lacerations but may also cause defects in the bones, joints, and tendons.

Ozdemir et al. have reported an average work time loss of 2.7 months after chainsaw-related lower-extremity injuries [1]. In the current study, we found an average work time loss of 57.89 days. This may be due to the fact that femur and tibia fractures were reported in their work, which were not present in our study. The treatment and healing of open fractures of the tibia and femur after contaminated injuries such as chainsaw injuries takes a longer time. We believe that the difference in loss of work between our study and the previous study is related to the fact that there were only tarsal, metatarsal, or phalanx fractures in our patients, except for one with a fibula fracture.

Data relating to patients’ return to their pre-injury job after saw injuries have been rarely reported in the literature. In two previous studies, 8.2% and 2.4% of patients with chainsaw-related injuries in their lower extremities were not able to return to their pre-injury jobs [1,7]. In our study, all patients (except for one with multiple finger amputations) were able to return to their pre-injury activities.

In the literature, saw-related injuries have been commonly related to hand injuries [2,7,8,9]. In our study, hand injuries comprised 41% of all injuries. While table saws have been reported as the most common cause of hand injuries, chainsaw injuries have also been reported [2,8,9,11]. However, a table saw is stationary and typically does not cause lower-extremity injuries (unless the person somehow falls on the table saw). As such, lower-extremity injuries are usually caused by portable circular saws and chainsaws [11]. In our study, all injuries were caused by chainsaws.

Saw-related injuries may be occupational, non-occupational, or woodshop-related [1,2,8,9,10]. All injuries (upper and lower extremities) in the current study were non-occupational injuries occurring in rural areas. A previous study has reported no statistically significant difference in terms of injury type between occupational and non-occupational injuries [12]. The most commonly reported injury mechanism related to chainsaws is kickback-type injuries [2,5,13]. Kickback, a phenomenon in which the blade flings backward toward the operator, is the greatest hazard related to chainsaws, which can cause serious injuries to the operator [5,13]. Kickback-type injuries usually cause upper-extremity, head, or neck injuries. Notably, there were no kickback injuries in our patients. In our series, there were three injury mechanisms for the lower extremities. The wood slipping out from underfoot while being cut with the tip of the chainsaw was the most common injury mechanism, typically causing medial-side injuries. A direct hit after cutting the wood was another mechanism of injury, which also caused medial-side injuries. Holding the chainsaw on the lateral side while the saw was still running was a less common cause of injuries, causing lateral-side injuries. Upper-extremity injuries were caused by a direct hit with a chainsaw while holding the wood piece, and patients with hand injuries were not operators.

The effect of hand dominance on the injury side is controversial, especially regarding hand injuries [10]. For lower-extremity fractures, hand dominance is important as the operator holds the tubular hand grip with their non-dominant hand and the pistol grip with their dominant hand. In this holding position, the contralateral lower extremity of the dominant hand is positioned at the front side and is affected by injury. In their series, Haynes et al. [5] reported that 68% of injuries affected the left side; meanwhile, in our series, the rate of left-side injuries (79.6%) was higher. In our series, injuries of the left lower extremity and lateral-sided right lower extremity were seen in patients with dominant right hands, while injuries of the medial-sided right lower extremity were seen in patients with dominant left hands. We did not research hand dominance in patients with upper-extremity injuries as none of them were operators.

The fracture rate in chainsaw-related injuries has been reported as ranging from 9.5 to 47% [1,2,4]. In our series, the fracture rate was 36.2% (26.6% for lower and 50% for upper extremities). The lower extremity fracture rate in our series was similar to that in previous studies [1]. We believe that the higher upper extremity fracture rate in our series was observed for two reasons: (1) all of the upper-extremity injuries were non-operators (directly hit by the saw) and (2) the lower extremities were partially protected by shoes or clothes (even if they were not special protective shoes/clothes).

A previous study on lower-extremity saw-related injuries has reported the tibia as the most commonly fractured bone [1]. In our study, there were no tibia fractures, and the proximal phalanx (n: 12) and first metatarsal (n: 9) were most commonly fractured. Ozdemir et al. also reported thirty-eight muscle/tendon injuries and three tibialis anterior injuries [1]. In our series, there were 104 tendon injuries, where the EHL (n: 57) was the most affected tendon.

In the literature, operators have been subjected to saw-related hand injuries [1,2,3,4]. In contrast, in our series, the patients with hand injuries were not operators, as all of them were holding wood. Sritharen et al. have reported skin lacerations as the most common injury type (68.9%); however, in our series, extensor tendon injuries were the most common injuries [2]. There were only three patients with isolated skin lacerations. The amputation rate was also higher (10.5%) in our study compared to the literature (6.7%).

To the best of our knowledge, no study has previously compared chainsaw-related upper- and lower-extremity injuries, and there are no previous data on the number of surgeries required after chainsaw-related injuries. In our study, upper-extremity injuries required statistically significantly more surgeries than lower-extremity injuries, which may be related to the higher fracture rate and number of amputations in the upper extremities.

Kim et al. have reported that the number of injuries increased each year over their four-year study period [12]. In our series, the number of chainsaw-related injuries increased from 2012 to 2015 and decreased from 2017 to 2020. The highest number of injuries were observed in 2021. Kim et al. also reported that upper-extremity injuries caused by power tools were significantly higher in May, April, and October [12]. In another study, Gemci et al. reported a higher incidence of injuries in January and November [7]. In the current study, both upper- and lower-extremity injuries were significantly higher in the autumn, especially in October (Figure 3). The residents of rural areas in our city still use wood for heating purposes in the winter; as such, in autumn, these people cut wood for winter storage. We believe that this is the reason for the higher number of injuries in this season.

We believe that chainsaw-related injuries, especially lower-extremity injuries, should be thoroughly debrided. In our experience, dust and wood pieces, as well as shoe, sock, and/or clothing pieces, were debrided in the current series. If possible, debridement should be performed in the operating theatre under anesthesia, as a sloppy debridement may cause serious infections later.

Chainsaw-related injuries overwhelmingly affect males [1,2,3,7]. In our series, there were only four female patients with hand injuries, none of which were operators.

Chainsaw-related injuries can be prevented through the use of proper safety equipment and proper chainsaw technique. To prevent injuries caused by chainsaws, protective equipment, such as specially designed gloves, shoes, and glasses, should be worn [1,14]. Instead of using the tip of the chainsaw to cut, the proximal part of the saw should be used to reduce the risk of slipping the saw over the wood. Furthermore, wood should not be held with the hand and fixed with the foot while cutting.

There are several limitations to our study. The main limitation of our study is its retrospective design. However, we gathered complete data for each of the patients, including the affected side and structures in detail, hand dominance, seasonal variations, being an operator or not, and loss of work time. The limited number of patients and short follow-up time are other limitations. Contrary to previous studies, which usually consider only lower- or upper-extremity injuries in isolation, the inclusion of both lower- and upper-extremity injuries is considered to be a valuable aspect of our study.

## 5. Conclusions

Chainsaws may cause serious injuries, most of which can be prevented by using the device carefully and cautiously. Upper-extremity injuries cause more loss of work time than lower-extremity injuries in the context of non-occupational chainsaw injuries.

## Figures and Tables

**Table 1:** Demographic data of patients.

Number of patients	185
Male	181
Female	4
Mean age of patients (years)	45.5 (17–81)
Mean follow-up (months)	9.3 (6–24)
Affected side	
Left	145
Right	40
Affected extremity	
Lower	109
Upper	76
Seasons	
Summer	31
Autumn	103
Winter	26
Spring	25
Length of hospital stay (days)	
Upper extremity	2.13 (1–10)
Lower extremity	2.39 (1–15)

**Table 2:** Statistics relating to lower- and upper-extremity injuries.

	Lower Extremity	Upper Extremity	*p*-Value
Age (years)	44.422 ± 13.952 (17–80)	47.329 ± 15.303 (18–81)	0.230 *
Length of stay (days)	2.037 ± 1.347 (1–10)	2.539 ± 2.506 (1–15)	0.201 **
Number of operations	1.019 ± 0.136 (1–2)	1.16 ± 0.466 (1–3)	0.004 **
Work time loss (days)	57.89 ± 24.517 (20–150)	75.197 ± 3 5.142 (20–270)	<0.001 **

*: Independent-samples *t*-test; **: Mann–Whitney U-test.

**Table 3:** Lower-extremity injuries and treatments.

Fractures and Partial Bone Damage (Chipping)	Number(s)	Treatment
Proximal phalanx	12	K-wire fixation
Middle phalanx	1	K-wire fixation
Distal phalanx	3	K-wire fixation
Metatarsal	9	4 K-wire fixation 5 conservative (for chipping injuries)
Cuneiform	1	Conservative
Talus	1	Conservative
Navicular	1	Conservative
Fibula	1	Plate and screw fixation
**Tendon injuries**		
Extensor hallucis longus	57	56 primary repair One EDC transfer
Extensor hallucis Brevis	10	Primary repair
Tibialis anterior	13	Primary repair
Tibialis posterior	6	Primary repair
Flexor hallucis longus	3	Primary repair
Extensor digitorum communis	5	Primary repair
Achilles	6	Primary repair
Peroneus longus and brevis	1	Primary repair
Patellar tendon	2	Primary repair
**Neurovascular injuries**		
Tibialis posterior artery	4	2 primary repair 2 ligated
Dorsalis pedis artery	2	Primary repair
Anterior tibial artery	1	Primary repair
Posterior tibial nerve	6	5 primary repair One was not suitable for repair
Superficial peroneal nerve	3	Primary repair
Deep peroneal nerve	2	Primary repair
Sural nerve	1	Not repaired

**Table 4:** Upper-extremity injuries and treatments.

Fractures and Partial Bone Damage (Chipping)	Structures Injured (n)	Treatment
Proximal phalanx	14	8 K-wire fixation 4 fixed with screws 2 conservative
Middle phalanx	7	6 K wire fixation 1 screw fixation
Distal phalanx	10	5 K wire fixation 1 screw fixation 4 conservative
Metacarpal	9	5 K-wire fixation 4 conservative
Scaphoid	1	Conservative
Radius	1	Conservative
**Tendon injuries**		
Extensor digitorum communis	24	Primary Repair
Extensor indicis proprius	3	Primary Repair
Extensor pollicis longus	4	Primary Repair
Extensor digiti minimi	3	Primary Repair
Extensor carpi radialis longus	1	Primary Repair
Extensor carpi radialis brevis	1	Primary Repair
Flexor digitorum superficialis	11	Primary Repair
Flexor digitorum profundus	9	Primary Repair
Flexor pollicis longus	2	Primary Repair
Flexor carpi radialis	5	Primary Repair
Flexor carpi ulnaris	3	Primary Repair
Brachioradialis	1	Primary Repair
**Neurovascular injuries**		
Digital artery	9	4 primary repair 5 ligated (not suitable for repair)
Ulnar artery	5	Primary Repair
Radial artery	2	Primary Repair
Digital nerve	11	10 primary repair 1 repaired with nerve graft
Ulnar nerve	5	Primary Repair
Radial nerve	2	Primary Repair
Median nerve	1	Primary Repair

## Data Availability

The data presented in this study are available on request from the corresponding author.
